# Supplementary material for: The exoS, exoT, exoU and exoY Virulotypes of the Type 3 Secretion System in Multidrug Resistant Pseudomonas aeruginosa as a Death Risk Factor in Pediatric Patients
Source: Pathogens. 2024 Nov 22;13(12):1030. doi: 10.3390/pathogens13121030 (PMC11677868; doi:10.3390/pathogens13121030)
Supplement: Supplementary file 1 [file pathogens-13-01030-s001.zip › Figure S1.pdf]

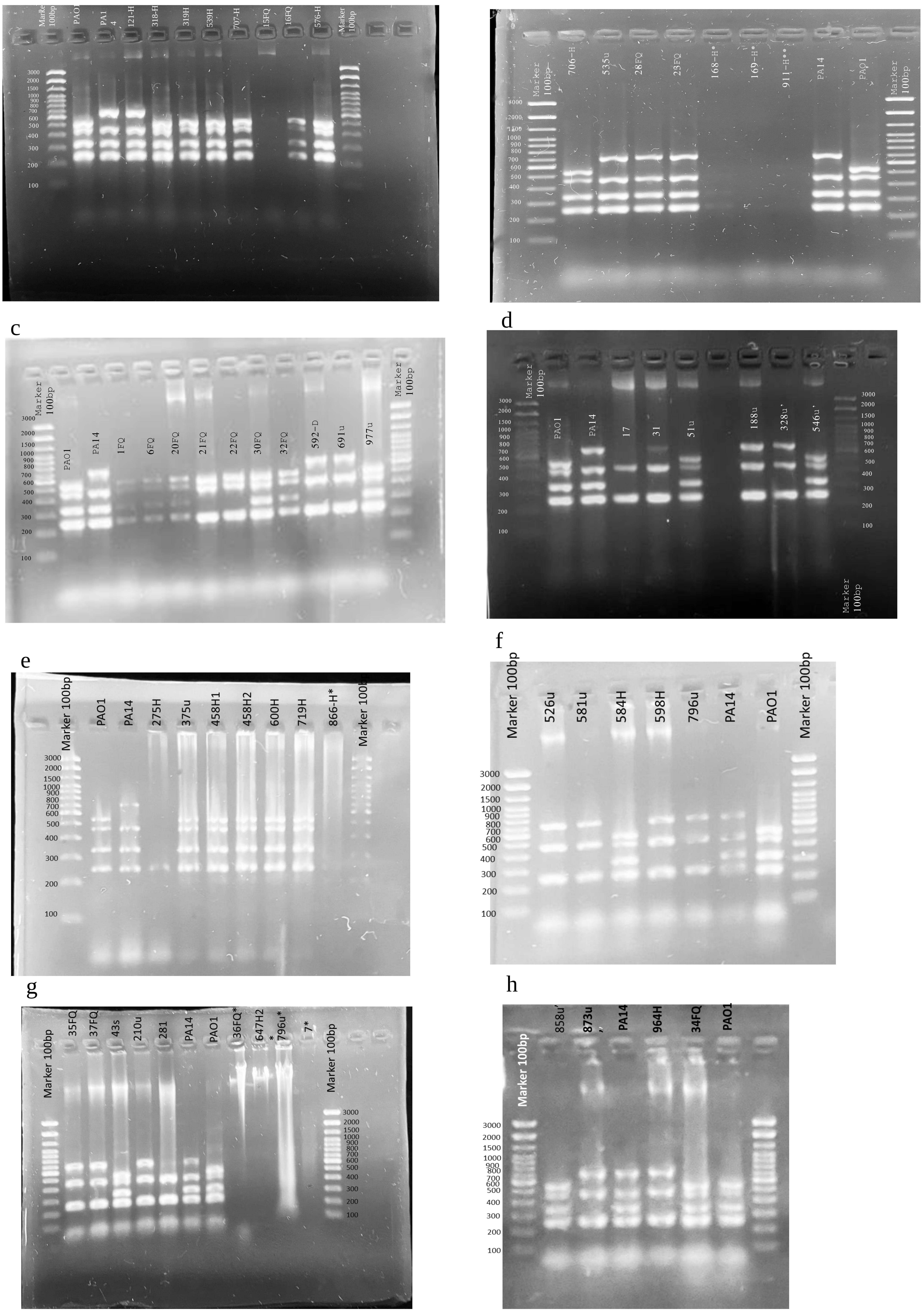

i

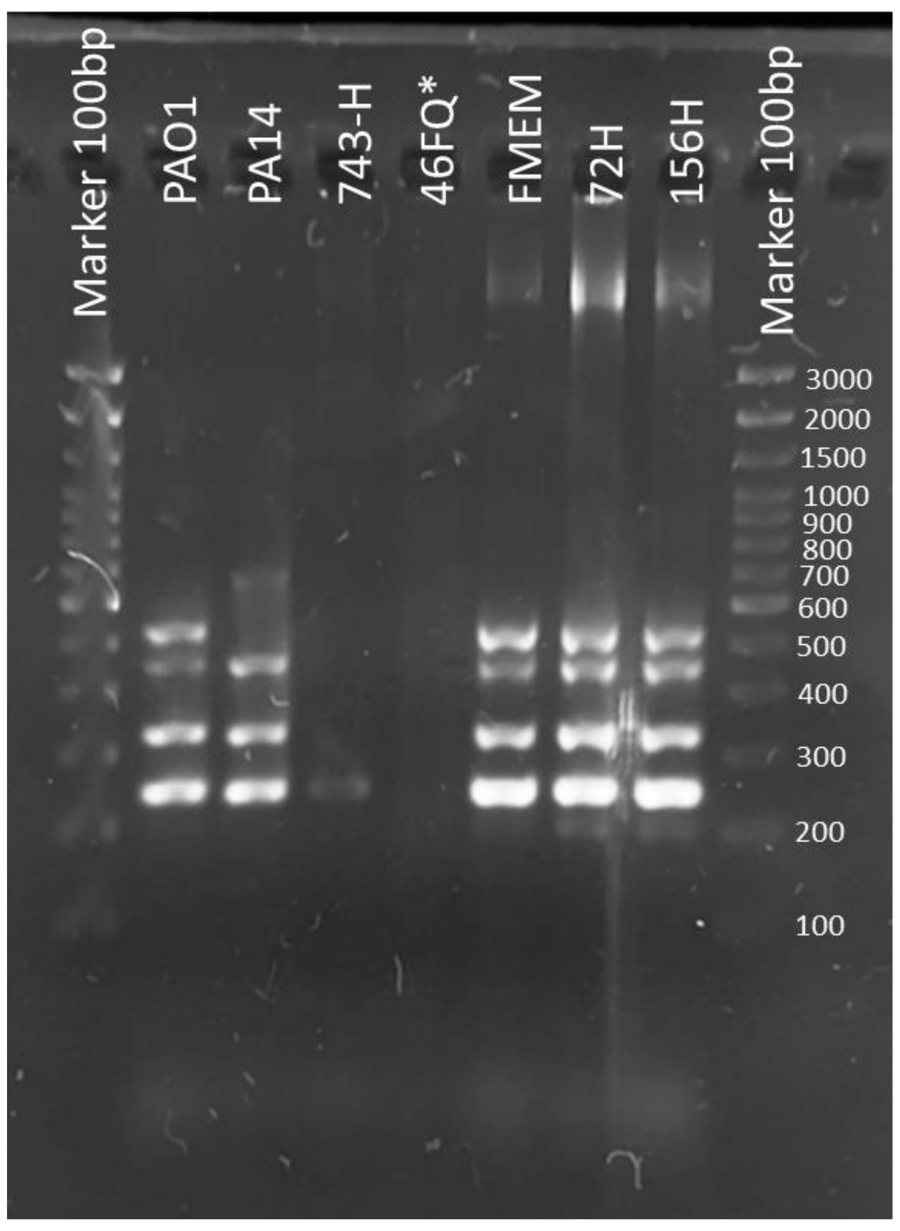

Figure S.1 Agarose gel electrophoresis of amplicons obtained by multiplex PCR using primer pairs for *exoU* (amplicon 688bp), *exoS* (amplicon 533bp), *exoT* (amplicon 459bp), *exoY* (amplicon 330bp) and *gyrB* (amplicon 243bp) genes. Positive control: PAO1 *exoS* and PA14 *exoU*, *negative control Samples: 15FQ *S. aureus, (a);* 168-H and 169-H *Stenotrophomonas maltophilia*, and 911-H** *Acinetobacter baumannii (b);* 866-H *Klebsiella pneumoniae (e);* 36FQ, 647H2 and 796u *Escherichia coli (g)* 46FQ *Staphylococcus aureus (i).*

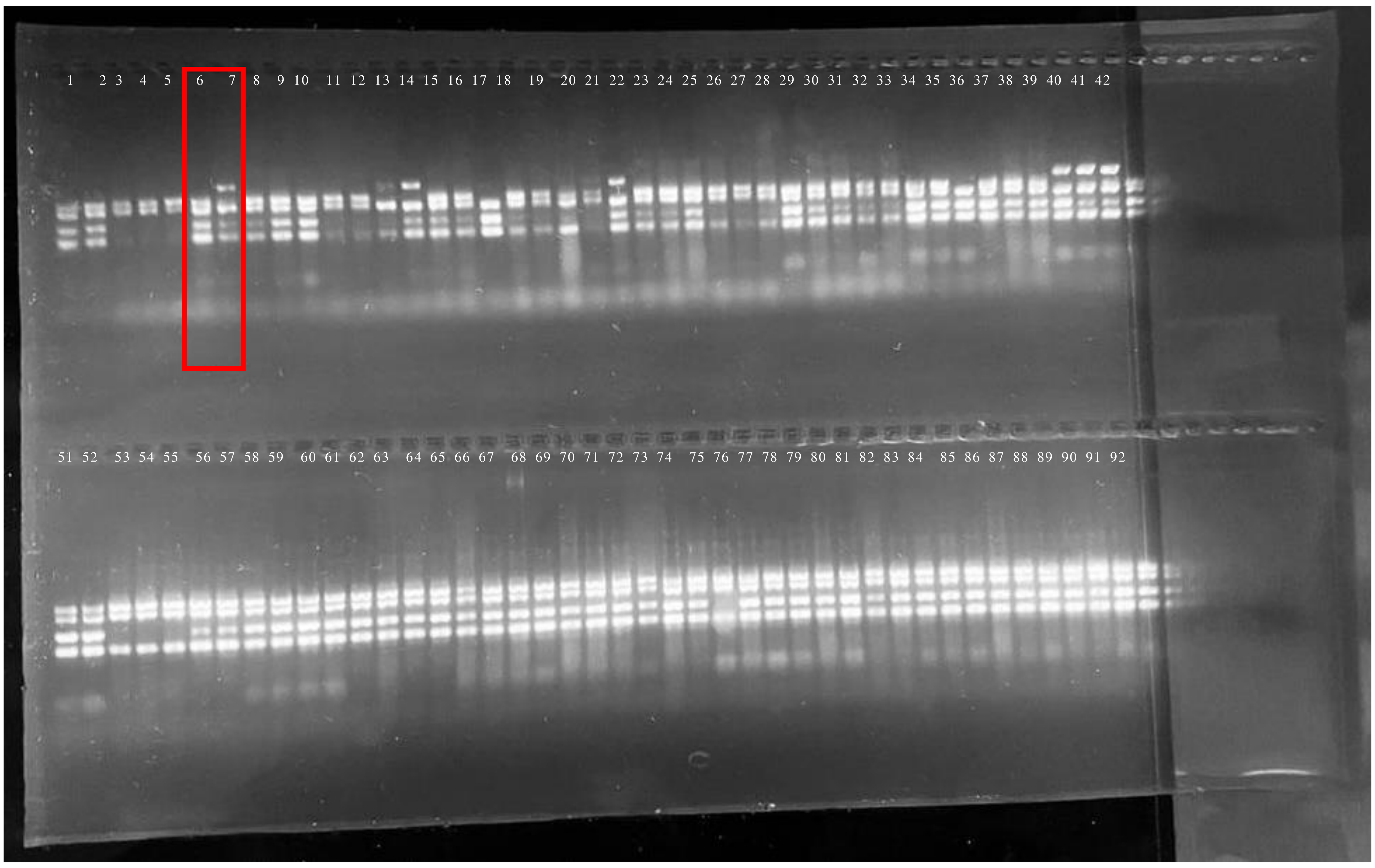

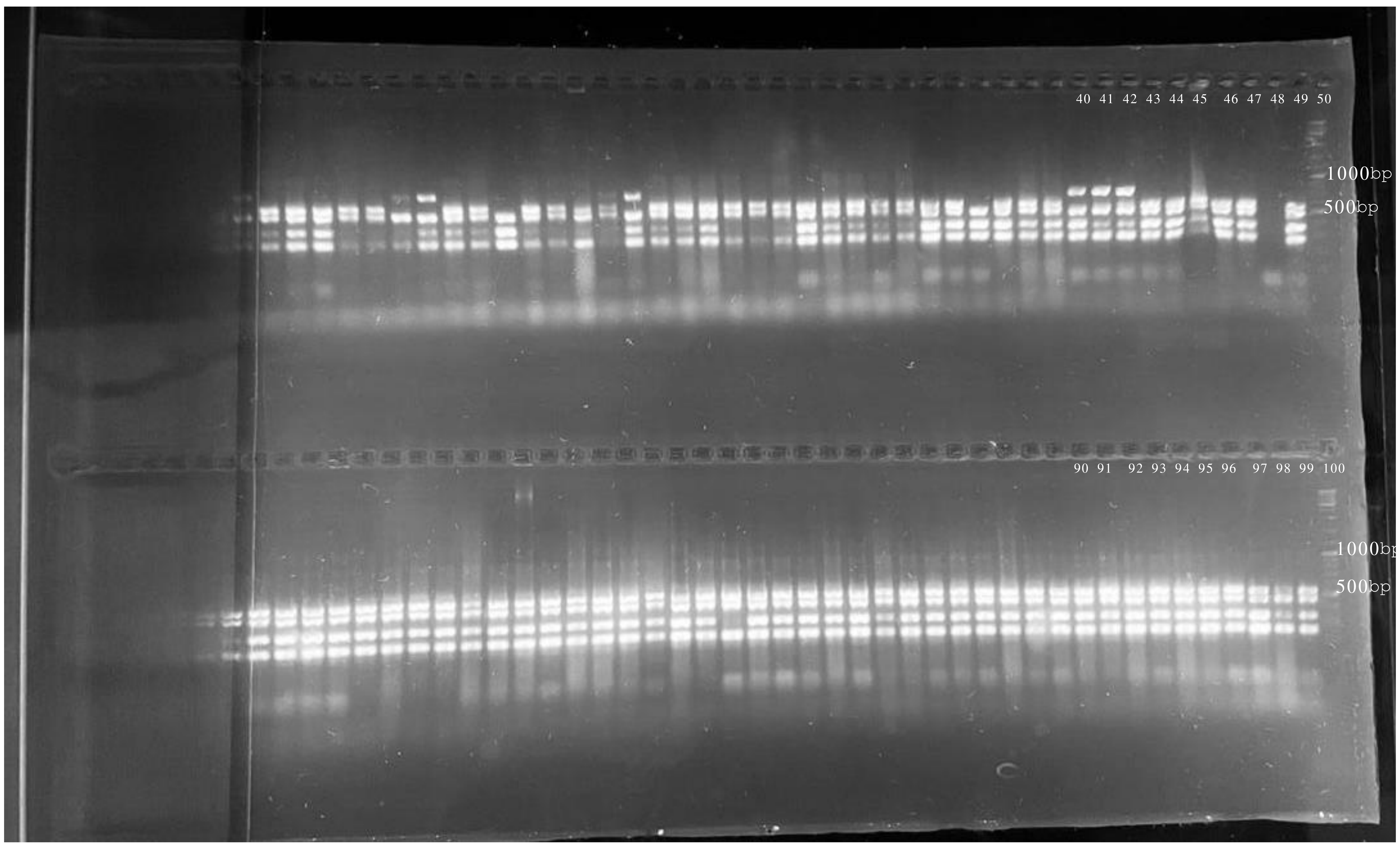

Figure S.1 Agarose gel electrophoresis of amplicons obtained by multiplex PCR using primer pairs for exoU (amplicon 688bp), exoS (amplicon 533bp), exoT (amplicon 459bp), exoY (amplicon 330bp) and gyrB (amplicon 243bp) genes. Tue positive controls PAO1 and PA14 are indicated in the red box. Lane: Sample: 1: 01-H; 2: 9 H, 3: 16-H; 4: 40-H; 5: 52; 6: PAO1; 7: PA14; 8: 120-H; 9: 135-H; 10: 136-H; 11: 137-H; 12: 138-H, 13: 139-H; 14: 140-H; 15: 192-H; 16: 193-H; 17: 197-H; 18: 207-H; 19: 221-H; 20: 222-H; 21: 229-H; 22: 230-H; 23: 231-H; 24: 237-H; 25: 239-H; 26: 240-H; 27: 241-H; 28: 253-H; 29: 265-H; 30: 257-H; 31: 258-H; 32: 259-H; 33: 260-H; 34: 263-H; 35: 269-H; 36: 302-H; 37: 336-H; 38: 337-H; 39: 338-H; 40: 344-H; 41: 348-H; 42: 349-H; 43: 369-H; 44: 377-H; 45: 388-H; 46: 389-H; 47: 404-H; 48: 409-H; 49: 413-H; 50: Marker 100bp; 51: 446-H; 52: 470-H; 53: 476-H; 54: 477-H; 55: 478-H; 56: 479-H; 57: 512-H; 58: 513-H; 59: 514-H; 60: 530-H; 61: 551-H; 62: 552-H; 63: 564-H; 64: 570-H; 65: 573-H; 66: 609-H; 67: 617-H; 68: 623_1; 69: 623_2; 70: 624-H; 71: 654-H; 72: 674-H; 73: 679-H; 74: 680-H; 75: 687-H; 76: 688-H; 77: 691-H; 78: 743-H; 79: 760-H; 80: 761-H; 81: 762-H; 82: 785-H; 83: 827-H; 84: 829-H; 85: 830-H; 86: 841-H; 87: 856-H; 88: 870-H; 89: 871-H; 90: 875-H; 91: 884-H; 92: 891-H; 93: 909-H; 94: 916-A; 95: 916-B; 96: 935-H; 97: 940-H; 98: 955-H; 99: 973-H; 100: Marker 100bp
