# Supplementary material for: The exoS, exoT, exoU and exoY Virulotypes of the Type 3 Secretion System in Multidrug Resistant Pseudomonas aeruginosa as a Death Risk Factor in Pediatric Patients
Source: Pathogens. 2024 Nov 22;13(12):1030. doi: 10.3390/pathogens13121030 (PMC11677868; doi:10.3390/pathogens13121030)
Supplement: Supplementary file 1 [file pathogens-13-01030-s001.zip › Table S2 Additional models.pdf]

Table S2 Additional models

Model 1
Type: Multinomial with Poisson Distribution
Response Variable: Virulotype
Predictor Variables: Death, Origin, ST1725, antibiotic susceptibility profile and haplotypes of the genes that regulate the MexAB-OprM pump (*mexR, nalC, nalD*).
Reference group: V3, MDR, non-ST1725, H1, non-Death, BSI

	**RR**	**p**	**CI(95%)**
**OV**			
Death	0.64	0.737	0.04-8.52
CF	4.86E-09	0.999	
PDR	10.50	0.221	0.242-455.701
XDR	8.11	0.060	0.91-72.02
S	5.47	0.366	0.13-217.94
ST1725	3.37	0.463	0.13-86.66
H0	1.54	0.769	0.085-27.61
H5	1.09	1	0
H8	3.35E-09	0.999	0
H12	0.58	0.739	0.02-13.57
H26	1.30E08	0.997	0
**V1**			
Death	1.62	0.707	0.13-20.15
CF	6.18E-09	0.999	0
PDR	34.98	0.095	0.53-2275.47
XDR	8.36	0.087	0.737-94.87
S	7.63	0.263	0.217-268.49
ST1725	0.66	0.799	0.02-15.88
HO	0.866	0.922	0.04-15.08
H5	2.45E09	0.999	0
H8	1.45E-09	0.998	0
H12	6.46E-09	0.997	0
H26	1.43E08	0.997	0
**V2**			
Death	2.66	0.411	0.25-27.67
CF	1.46E-07	0.999	0
PDR	34.98	0.095	0.53-2275.473
XDR	8.36	0.087	0.737-94.87
S	7.63	0.263	0.21-268.493
ST1725	0.66	0.799	0.027-15.88

HO	0.86	0.922	0.049-15.083
H5	2.94E09	0.999	0
H8	1.45E-09	0.998	0
H12	6.46E-09	0.997	0
H26	1.43E08	0.997	0

RR: relative risk, *: significant *p* value <0.05, CI: confidence interval = 95%, CF: cystic fibrosis, BSI: blood-stream infection, PDR: pandrug resistant, XDR: extensively drug resistant, S: sensitive. V1: *exoU+/exoS-/exoT+/exoY+;* V2: *exoU+/exoS-/exoT+/exoY-;* V7: *exoU-/exoS+/exoT+/exoY-*; OV: other-virulotype group. Conformed by V4 (*exoU-/exoS-/exoT+/exoY-)*, V5 (*exoU-/exoS-/exoT+/exoY+)*, V6 (*exoU-/exoS-/exoT-/exoY-),* V8 (*exoU-/exoS-/exoT-/exoY+)*, V9 (*exoU+/exoS+/exoT+/exoY+)*, V10 (*exoU-/exoS+/exoT-/exoY+),* and V11 (*exoU+/exoS+/exoT+/exoY-).*

<u>Model 2</u>
Type: Binomial
Response Variable: ST1725
Predictor Variables: Virulotype, Death, Origin and antibiotic susceptibility profile
Reference group: OV, MDR, non-Death , BSI

ST1725	RR	P	CI95%
CF	0	0.9959	0
V1	0.4439	0.3866	-2.74-0.9908
V2	7.3761	0.0178*	0.4199-3.7969
V3	0.0622	0.0057*	-5.031 - -0.9717
Death	0.3721	0.2479	-2.7963 – 0.0350
PDR	30.4273	0.0069*	1.0200 – 6.1060
S	0	0.9938	0
XDR	4.0002	0.2390	-0.8315 – 3.8964

RR: relative risk, *: significant *p* value <0.05, CI: confidence interval = 95%, CF: cystic fibrosis, PDR: pandrug resistant, XDR: extensively drug resistant, S: sensitive. V1: *exoU+/exoS-/exoT+/exoY+;* V2: *exoU+/exoS-/exoT+/exoY-;* V3: *exoU-/exoS+/exoT+/exoY+).*

<u>Model 3</u>
Type: Binomial
Response Variable: H12
Predictor Variables: Virulotype, Death, Origin and antibiotic susceptibility profile
Reference Group: OV, MDR, non-Death, BSI

H12	RR	P	CI95%
CF	0	0.9991	-4.4091 – 0.1208
V1	0	0.9968	
V2	0	0.9946	
V3	1.6406	0.5621	
Death	0.4603	0.5582	-3.9745 – 1.7037
PDR	0	0.9954	
S	0	0.9986	
XDR	7.0341	0.0329*	0.3030 – 4.0245

RR: relative risk, *: significant *p* value <0.05, CI: confidence interval = 95%, CF: cystic fibrosis, PDR: pandrug resistant, XDR: extensively drug resistant, S: sensitive. V1: *exoU+/exoS-/exoT+/exoY+;* V2: *exoU+/exoS-/exoT+/exoY-;* V3: *exoU-/exoS+/exoT+/exoY+).*
